# Molecular characterization of intergeneric hybrids between *Malus* and *Pyrus*

**DOI:** 10.1093/hr/uhac239

**Published:** 2022-10-26

**Authors:** Giulia Pasqualetto, Luisa Palmieri, Stefan Martens, Vincent G M Bus, David Chagné, Claudia Wiedow, Mickael A Malnoy, Susan E Gardiner

**Affiliations:** Research and Innovation Centre, Edmund Mach Foundation, San Michele all'Adige, TN 38010, Italy; Department of Agricultural, Food, Environmental and Animal Sciences, University of Udine, UD 33100, Italy; The New Zealand Institute for Plant and Food Research Ltd (PFR), Hawke’s Bay Research Centre, Havelock North, New Zealand; Research and Innovation Centre, Edmund Mach Foundation, San Michele all'Adige, TN 38010, Italy; Research and Innovation Centre, Edmund Mach Foundation, San Michele all'Adige, TN 38010, Italy; The New Zealand Institute for Plant and Food Research Ltd (PFR), Hawke’s Bay Research Centre, Havelock North, New Zealand; PFR, Fitzherbert Science Centre, Palmerston North, New Zealand; PFR, Fitzherbert Science Centre, Palmerston North, New Zealand; Research and Innovation Centre, Edmund Mach Foundation, San Michele all'Adige, TN 38010, Italy; PFR, Fitzherbert Science Centre, Palmerston North, New Zealand

## Abstract

Apple (*Malus*) and pear (*Pyrus*) are economically important fruit crops well known for their unique textures, flavours, and nutritional qualities. Both genera are characterised by a distinct pattern of secondary metabolites, which directly affect not only resistance to certain diseases, but also have significant impacts on the flavour and nutritional value of the fruit. The identical chromosome numbers, similar genome size, and their recent divergence date, together with DNA markers have shown that apple and pear genomes are highly co-linear. This study utilized comparative genomic approaches, including simple sequence repeats, high resolution single nucleotide polymorphism melting analysis, and single nucleotide polymorphism chip analysis to identify genetic differences among hybrids of *Malus* and *Pyrus*, and F2 offspring. This research has demonstrated and validated that these three marker types, along with metabolomics analysis are very powerful tools to detect and confirm hybridity of progeny derived from crosses between apple and pear in both cross directions. Furthermore, this work analysed the genus-specific metabolite patterns and the resistance to fire blight (*Erwinia amylovora*) in progeny. The findings of this work will enhance and accelerate the breeding of novel tree fruit crops that benefit producers and consumers, by enabling marker assisted selection of desired traits introgressed between pear and apple.

## Introduction

The subfamily of Pomoideae (family Rosaceae) comprises a number of genera known as “pome fruits”, which are valuable fruit crops for human nutrition and health [1]. Apple (*Malus* x *domestica* Borkh.) is the major crop with respect to global consumption, followed by pear (*Pyrus communis* L., *P. bretschneiderii* Rehder, *P. pyrifolia* (Burm.f.) Nakai) and quince (*Cydonia oblonga* Mill.) [2].

Apple and European pear differ in texture, flavour, and nutritional qualities. Each has a specific pattern of secondary metabolites, that impacts fruit flavour and nutritional value as well as affecting resistance to horticulturally significant diseases [3, 4]. They have an identical chromosome number [5, 6], with a recent divergence date [7] and DNA marker analysis has demonstrated that their genomes are highly co-linear [8].

Intergeneric hybrids between apple and pear provide a unique germplasm resource for developing novel pome fruits, in breeding programmes supported by early-stage genomic and metabolic profiling. Interspecific hybridization is acknowledged as the most important source of genetic variation for breeding new varieties [9]. Interspecific hybrids can be used to develop new morphological forms of plants and fruit, as well as combining characteristics of two taxa into one and are being investigated for development of new rootstocks for the citrus industry [10]. Although interspecific crosses have been employed in pear breeding for decades, with crosses among Asian (*P. bretschneideri*, *P. pyrifolia*) and European pear (*P. communis* L.) used to develop novel progeny [11]. However, attempts to overcome the intergeneric crossing barriers between *Malus* and *Pyrus* have been infrequent. Successful intergeneric crosses would produce hybrids enabling the introduction of various chromosomal regions of the pear genome into *Malus* via subsequent backcrosses with *Malus*, or *vice versa* and hence the introgression of flavour, texture, resistances and other important quality and agronomic traits into the progeny [12].

The first intergeneric hybrids between pears and apples were reported by Crane and Marks [13] (1952). An apple x European pear hybrid obtained in 1970 had intermediate phenotype. The fruit were pear-shaped and seeds were rare, but when present appeared viable. Fruit yield was poor, the pollen was sterile and after a few years the hybrid died [14]. Inoue *et al*. [15] reported that lethality in hybrids between Japanese pear (*P. pyrifolia*) and apple was suppressed at high temperatures (34/30°C, day night, or at constant 34°C). Death of the intergeneric seedlings within five months was presumed to have been caused by physical stress from high temperature.

In a study of hybrids between Japanese pear and apple, where hybrid embryos were gamma-irradiated and cultured at normal temperatures, to obtain viable intergeneric offspring, Gonai *et al*. [16] reported that embryos were mostly aborted at an early developmental stage, or seedlings died within six months. In 2014, Zwintzscher’s Hybrid, which resulted from a cross between *Malus* and *Pyrus* by Max Zwintscher was reported to be intergeneric [12], using S-allele and DNA content, as well as metabolomics analyses. This F1 hybrid was grafted and maintained by Hermann Schimmelpfeng and has given rise to a fertile F2 generation following open pollination. However, no systematic attempt had been made previously to generate crosses in both directions and the number of progenies were low in all these previous studies. Furthermore, no progeny with pear as the female parent had survived long term.

We describe our generation of new intergeneric progeny between apple and European pear, from crosses made in both directions and pioneered comparative genomics approaches to identify genetic differences among 66 new putative intergeneric *Malus* x *Pyrus* hybrids, as well as their offspring. The use of genomics provided insight into the genetic reorganization of the hybrids, through mapping the genomic segmentation between apple and pear in their progeny. Furthermore, this work also analysed the genus-specific metabolite patterns exhibited in the intergeneric hybrids, using arbutin as indicative of *Pyrus* and phloridzin as indicative of *Malus* among the intergeneric hybrids. Additionally, hybrids were also assessed for resistance to fire blight (*Erwinia amylovora* (Burrill) Winslow, Broadhurst, Buchanan, Krumwiede, Rogers & Smith), a disease that is significant in pome fruit production internationally [17], as a preliminary trial to test whether introgression of resistance between the genera might be possible. The findings of this work will accelerate the breeding and development of novel tree fruit crops, by enabling the marker assisted selection of seedlings carrying desirable traits introgressed between pear and apple.

## Results

### Development of intergeneric crosses

See Supplementary Results.

### SSR (simple sequence repeat) genotyping of the controls and putative intergeneric hybrids

**Figure 1:**
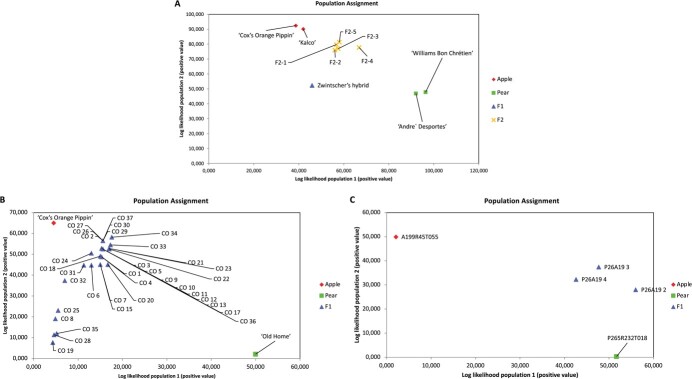
Population assignment of putative intergeneric apple/pear hybrids as deduced from the simple sequence repeat (SSR) marker analysis. The plots represent the positive log-likelihood of assignment of each sample by GenAlEx. The log-likelihood values calculated assigned the apple parents (“Cox’s Orange Pippin”; A199R45T055; “Kalco”) to one group/population, the pear parents (“Old Home”; P265R232T018; “André Desportes”; “Williams Bon Chrétien”) to a second group/population, and the putative hybrids to a cluster between these two groups. Figure 1A shows the Fondazione Edmund Mach (FEM) population, 1B “Cox’s Orange Pippin” x “Old Home” population and 1C the P265R232T018 x A199R45T055 population.

Of the 34 SSR markers screened over the Plant & Food Research (PFR) and Fondazione Edmund Mach (FEM) populations, 21 and 30 were informative (heterozygous), respectively. The GenAlEx software assignment, which uses parental data to position the progeny, demonstrated the validity of the SSR method for detecting hybrids, confirming that the control Zwintzscher’s Hybrid was a true F1 intergeneric hybrid (Fig. 1A). The apple (“Cox’s Orange Pippin”; A199R45T055; “Kalco”) and pear (“Old Home”; P265R232T018; “André Desportes”; “Williams Bon Chrétien” (“Williams Christ”) parents clustered in different groups (Figs 1A, 1B and 1C).

**Figure 2:**
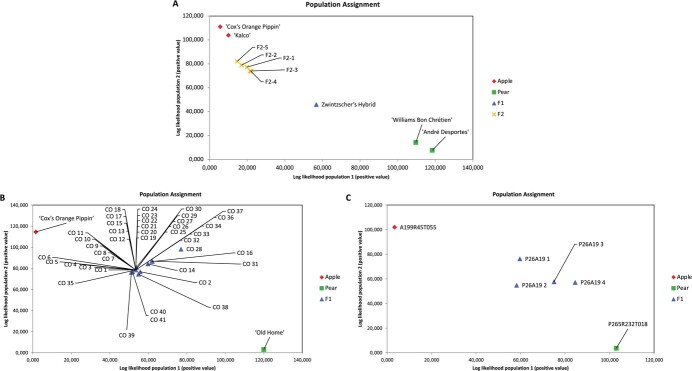
Population assignment of putative intergeneric apple/pear hybrids as deduced from the high resolution melting (HRM) marker analysis. The plots represent the positive log-likelihood of assignment of each sample by GenAlEx. The log-likelihood values calculated assigned the apple parents (“Cox’s Orange Pippin”; A199R45T055; “Kalco”) to one group/population, the pear parents (“Old Home”; P265R232T018; “André Desportes”; “Williams Bon Chrétien”) to a second group/population, and the putative hybrid to both two groups. Figure 2A shows the Fondazione Edmund Mach (FEM) population, 2B the “Cox’s Orange Pippin” x “Old Home” population and 2C the P265R232T018 x A199R45T055 population.

The five Zwintzscher’s Hybrid F2 open pollinated progeny (Fig. 1A) grouped between the two parental clusters, closer to apple than pear, while the putative intergeneric hybrids from PFR, (35 “Cox’s Orange Pippin” x “Old Home” (CO) F1 progeny) clustered between apple and pear, with GenAlEx assignment values ranging from 0.60, for CO 35, to 5.89 for CO 32 (Fig. 1B). Log-likelihoods values were converted to positive values to facilitate data presentation. The lowest value on the X and Y axes indicates the most likely position relative to apple and pear, for each sample, respectively.

CO hybrids were positioned closer to the apple maternal parent than pear, demonstrating they are true apple-pear hybrids, but related more closely to apple than pear. However, six CO putative hybrids appeared to group separately, having low assignment values for both apple and pear. The three P265R232T018 x A199R45T055 progeny (Fig. 1C) exhibited assignment values characteristic of true hybrids, and clustered closer to the pear female than to apple.

The discrimination power and the robustness of the SSR markers used were assessed by genetic diversity analysis (Supplementary Table 1).

### High resolution melting (HRM) analysis

Of the 155 HRM markers screened over the parents and subset of hybrids, 39 were informative. Of these, 36 markers demonstrated that the HRM method was valid for detecting hybridity, as the control Zwintzscher’s Hybrid exhibited a double melting peak amplicon indicative of heterozygosity. Furthermore, one to six HRM markers per genotype provided the same evidence for hybridity of the five Zwintzscher’s Hybrid F2 progeny. Following GenAlEx population assignment analysis, the F1 and F2 hybrids were positioned between the apple and pear parental clusters, with Zwintzscher’s Hybrid located centrally and the F2 hybrids closer to the apple parents (Fig. 2A). Of the PFR putative hybrids, the 41 CO apple-pear progeny showed evidence of hybridity, with 9 to 15 HRM markers per accession (Fig. 2B). Eight to 30 HRM markers per genotype provided evidence for hybridity of the four pear-apple hybrids between P265R232T018 x A199R45T055 (Fig. 2C).

As observed for the SSR markers, the GenAlEx population assignment analysis using the informative HRM marker data clustered the apple and pear parents to separate groups on the X and Y axes. Zwintzscher’s Hybrid, all 41 putative apple-pear hybrids from the CO progeny, and all four putative P265R232T018 x A199R45T055 (P26A19) were located between the two parent groups (apple and pear), and the five Zwintzscher’s Hybrid F2 progeny were located near apple, between Zwintscher’s Hybrid and apple (Fig. 2A). The discrimination power (PD) and the robustness of the HRM markers used were assessed by the genetic diversity analysis. The extreme values of all parameters analysed for each population indicate that the genetic diversity of the New Zealand and FEM populations is very similar (Supplementary Table 2).

### Single nucleotide polymorphism (SNP) array genotyping of intergeneric hybrids

In total, 1090 apple SNPs were polymorphic in the CO progeny, after filtering of the apple and pear Infinium® II 9 K SNP array data. No pear SNPs were retained after filtering, owing to the presence of null alleles in parents or progeny.

GenAlEx population assignment analysis using the SNP-chip marker data clustered the apple, and pear parents (“Cox’s Orange Pippin” and “Old Home”) to separate groups on the X and Y axes (Supplementary Figs. 1, 2, 3 and 4). The putative hybrids grouped between the two parental clusters. The GenAlEx population analysis showed that 26 F1 samples, as well as CO 7 and CO 26 exhibited the same results and apparently indicating they are identical. However, this finding did not agree with the SSR and HRM analyses. Two other progenies (CO 16 and CO 37) were not identical.

**Figure 3:**
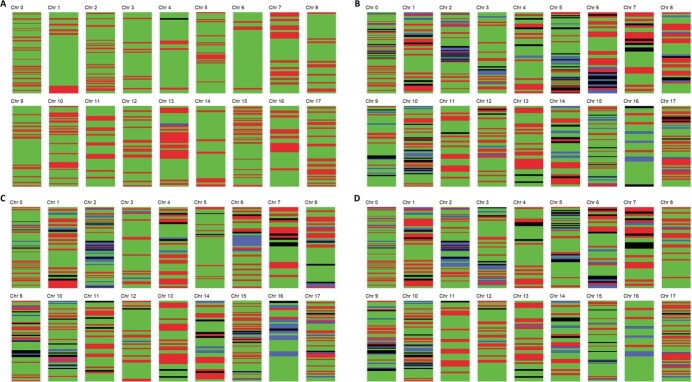
Mapping of genomes of the “Cox’s Orange Pippin” x “Old Home” population with the 9 K apple-pear SNP array by chromosome for the apple SNPs. The pear SNPs were all placed in one group, Chr0. Areas in green indicate that SNP results support the hybridity of the F1, red when the results were the same as the mother, blue when the results were the same as the father, and black when the progeny have different results to both mother and father. A represents the map of CO 2, CO 3, CO 4, CO 5, CO 9, CO 10 CO 11, CO 12, CO 13, CO 14, CO 15, CO 17, CO 19, CO 20, CO 22, CO 23, CO 24, CO 25, CO 27, CO 29, CO 31, CO 33, CO 34, CO 35, CO 40; B represents the map of CO 7 and CO 26; C CO 16 and D for CO 37.

In these two non-duplicated individuals, 692 SNP markers (63.5%) supported hybridity in one individual and 682 (62.6%) in the other. Fig. 3 represents the four genomic segmentation maps constructed for representative progeny from population CO: one of the 26 F1 progeny samples in Fig. 3A; CO 7 and CO 26 in Fig. 3B; CO 16 in Fig. 3C; and CO 37 in Fig. 3D. In this figure, regions with SNPs supporting hybridity are shown in green; these have heterozygous SNP genotypes, and the alternative alleles were homozygous in the parents.

The sample mapped in Fig. 3A exhibited more SNPs supporting hybridity (75.6% heterozygous SNPs), than the other samples in Fig. 3 (B, C and D), where the hybridity is supported by respectively, 60.1% (B), 63.5% (C) and 62.6% (D) heterozygous SNPs (D). The percentage of SNPs apparently originating from the mother (in red) were 24.2% (3A), 24.3% (3B), 23.4% (3C) and 23.2% (3D), while only 0.1% (3A), 7.3% (3B), 6.9% (3C) and 7.4% (3D) originated from the father (in blue). Furthermore, in black, 0.1% (A), 8.2% (3B), 6.2% (3C) and 6.8% (3D) SNPs were completely different from both parents (data for all samples are summarised in Table 1).

**Table 1:** Percentage of single nucleotide polymorphisms (SNPs) for each progeny group in Fig. 3 supporting hybridity (in green), SNPs apparently originating from the mother (in red), SNPs apparently originating from the father (in blue) and SNPs completely different from both parents (in black). A represents the genome map of “‘Cox’s Orange Pippin”’ x “‘Old Home”’ (CO) 2, that is the same as those of CO 3, CO 4, CO 5, CO 9, CO 10 CO 11, CO 12, CO 13, CO 14, CO 15, CO 17, CO 19, CO 20, CO 22, CO 23, CO 24, CO 25, CO 27, CO 29, CO 31, CO 33, CO 34, CO 35, CO 40; B represents the map of CO 7 and CO 26; C represents CO 16 and D represents CO 37 (Fig. 3).

	A	B	C	D
SNPs supporting hybridity (in green)	75.6%	60.2%	63.5%	63.5%
SNPs apparently originating from the mother (in red)	24.2%	24.3%	23.4%	23.4%
SNPs apparently originating from the father (in blue)	0.1%	7.3%	6.9%	7.4%
SNPs completely different from both parents	0.1%	8.2%	6.2%	6.2%

### Targeted metabolomics analysis of intergeneric hybrids

**Figure 4:**
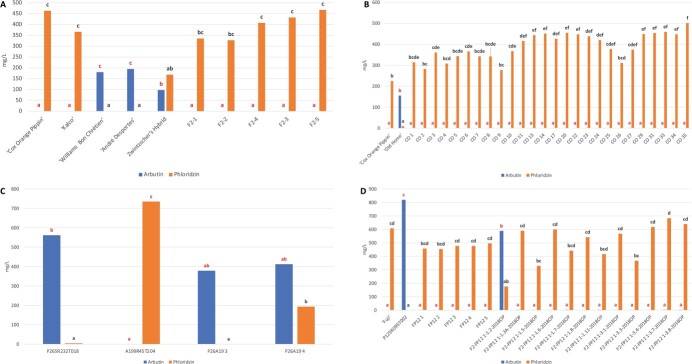
Mean concentrations of arbutin (blue) and phloridzin (orange) in: F2 apple/pear hybrids and their parent and (great) grandparents of the Fondazione Edmund Mach population (A); “Cox’s Orange Pippin”, “Old Home” and their progeny (B); P265R232T018, A199R45T055 and their progeny (C); “Fuji”, pear seedling R095, their progeny FP12, and their open-pollinated F2 offspring from FP12 1 (D).

Metabolomics analysis confirmed that Zwintzscher’s Hybrid, used as a positive control, accumulates metabolites typical of both pear and apple in its leaves, while two members of its pedigree, “Williams Bon Chrétien” (pear) accumulated arbutin specific to *Pyrus* and “Cox’s Orange Pippin” (apple) accumulated phloridzin typical of *Malus* (Fig. 4A). The concentration of phloridzin in the Zwintzscher’s Hybrid F2 progeny was considerably higher than that of arbutin (Fig. 4A).

Members of the CO population accumulated only phloridzin inherited from the female apple parent “Cox’s Orange Pippin” (Fig. 4B). Progeny P26A19 4 from the pear P265R232T018 x apple A199R45T055 cross accumulated high concentrations of metabolites typical of both pear (arbutin) and apple (phloridzin) in its leaves (Fig. 4C), while only arbutin from *Pyrus* was detectable in the sister seedling P26A19 3. P26A19 4 was the only F1 progeny from PFR confirmed to be an intergeneric hybrid based on the metabolomics analysis alone. All five FP12 progeny of female apple parent “Fuji” crossed with P125R095T002 and the subsequent F2-FP12–1 population accumulated phloridzin, with F2-FP12–1-1.2-OP accumulating significantly less than the others, and being the only progeny accumulating arbutin in significant amounts (Fig. 4D).

### Fire blight resistance assessment of intergeneric hybrids

**Figure 5:**
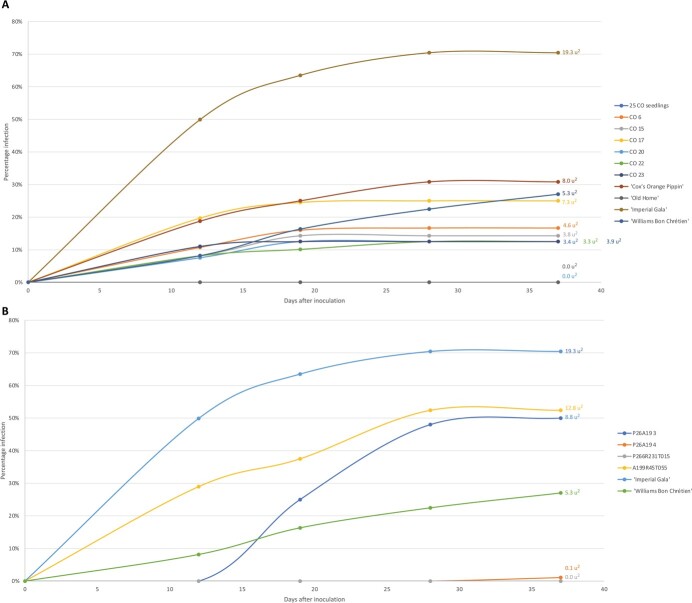
Fire blight necrosis progress and the value of area under the disease progress curves (AUDPC) in “Cox’s Orange Pippin” x “Old Home” population and reference accessions (A), P265R232T018 x A199R45T055 population and reference accessions (B) after inoculation with *Erwinia amylovora* using the cut-leaf method.

All 31 putative CO hybrids screened showed resistance to fire blight (Fig. 5A). It is noteworthy that the degree of observed fire blight resistance in all progenies was greater than that for the “Cox’s Orange Pippin” apple parent, which exhibited 31.5% necrosis at the final measurement. A total of 25 hybrids exhibited 0% necrosis, the same as for the pear parent “Old Home”, while the remaining six progeny showed 12.5% to 25.0% necrosis, and area under the disease progress curve (AUDPC) from 3.3 u^2^ to 7.3 u^2^ at the final measurement. In the more susceptible plants, necrosis length had reached its maximum by the 19^th^ day after inoculation (data not presented). Although “Imperial Gala” and “Williams Bon Chrétien” were both susceptible controls, their resistance profiles differed over time (Fig. 5A). At 28 days after inoculation, necrosis in “Imperial Gala” had plateaued, whilst for “Williams Bon Chrétien”, the necrosis was still increasing at 37. At 28 days after the inoculation, necrosis in “Imperial Gala”, A199R45T055 and P26A19 3 had plateaued, whilst in “Williams Bon Chrétien”, necrosis was still increasing at 37 days (Fig. 5B). With 1.1% necrosis and AUDPC of 0.1 u^2^ at the end of the assessment period, P26A19 4 exhibited a high level of resistance, similar to that of its pear parent P265R232T018. In contrast, with 50.0% necrosis and AUDPC of 8.8 u^2^ at the final measurement, P26A19 3 was susceptible, similar to its apple parent A199R45T055.

## Discussion

We have established a series of genetic and phenotypic technologies to identify pear-apple intergeneric hybrids and demonstrated the power of using several marker systems to screen them. Our intergeneric crosses in both directions, contribute an important and unique set of germplasm to researchers and breeders. No successful pear x apple crosses had been raised to fruiting previously [16]. The previously characterized intergeneric apple-pear hybrid Zwintzscher’s Hybrid served as a positive control for the validation of the methods we employed to characterize our new hybrids. Our markers included published SSRs developed fromapple and pear, HRM-based markers using primers developed from whole genome re-sequencing of apple and pear accessions, as well as SNP array technology. Analysis of the genus-specific metabolites arbutin (pear) and phloridzin (apple) proved to be a useful, fast and reliable tool to confirm hybridity of our new plant material, as was previously shown for the control Zwintscher’s Hybrid.

HRM markers appeared to be more informative than SSR markers, because almost all the HRM markers had a discrimination power that was low for the parent, but higher for the progeny, while having a low Shannon’s information index, as expected for a high quality marker (Supplementary Tables 1, 2). These findings are in agreement with Distefano *et al*. [18] for *Citrus.* The more robust assessment of hybridity using the HRM method can be attributed to the very distinct double-peaked melting profiles generated by heterozygous HRM amplicons [19]. Nevertheless, it is not the quickest to develop, because of the need to design HRM primers for the genetic background of the parents in crosses andto undertake pre-screen performance testing of parents. However, whole genome re-sequencing is now publicly available for an increasing number of pear and apple cultivars that might be used as parents [20, 21] and the cost of sequencing continues to decrease. This means that re-sequencing data with adequate coverage can be produced in an affordable and reproducible manner making the development of HRM markers from re-sequencing data more practical.

The SNP array analysis of the CO population confirmed the SSR and HRM results for hybridity in the CO progeny. An issue with the 9 K SNP array [22] used in this study was its balance between apple and pear SNPs, whereby there are 8 K apple SNPs, but only 1 K pear SNPs. Almost all pear SNPs were removed from the SNP dataset after filtering, due to the presence of null alleles in the progeny, as well as null allele results for both parents. The SNP array results may also have been limited in comparison with the HRM results, because the SNPs used for our SNP array analysis were specific to the plant material used in array design, rather than the material screened, making the pre-screened HRM markers more powerful than the SNP array in our hands.

Previous researchers have suggested that different methods may be preferred for different levels of analysis, SSRs being preferred at the level of the individual and SNPs at the population level. A comparative analysis between the capacity of SNPs and SSRs to investigate genetic variation in juniper (*Juniperus phoenicea* ssp. *turbinata* (Guss.) Parl.) demonstrated the higher per-locus information content of SSRs made them the marker of choice for parentage and assignment studies, whereas SNPs provided more reliable demographic inferences [23]. Singh *et al*. [24] suggested that resolution of populations of Indian rice varieties was higher with SNP markers, but SSRs were more efficient for diversity analysis. Zurn *et al*. [25] demonstrated the continued usefulness of SSRs for certain applications (e.g. diversity assessment and identity confirmation) and that SSRs are better at detecting population structure that may be missed when using bi-allelic SNPs. SNP-chip analyses are also very specific to the species used [26].

The SNP array screen of the CO population suggested that several samples (26 in one set and two others in a second set) were genetically identical, although our SSR and HRM analyses did not indicate the presence of replicates in the CO population. It is possible that during the *in vitro* culture some plantlets were replicated, however, we believe that our HRM results are more trustworthy, as the primers were designed for the parents of the CO population, while the plant material used in design of the SNP array was more distantly related to the CO progenies. Issues with our SNP array results might have arisen due to difficulties in hybridization of the apple x pear DNA to the array probes.

Some of the results for SNP segmentation mapping in the CO population did not agree with the predicted inheritance from both mother and father, in addition to the expected bi-allelic segments inherited from the female and male parents, there were also segments that appeared to be inherited from neither parent. Whilst a few of these events could be real, arising from anomalous events in the cell cycle due to the wide apple-pear cross [27], they might result from technical issues caused by primer mismatches arising from extra SNPs in the target sequence causing anomalous results during the array run. Similar observations were found using SSR analysis, where in a few instances there were additional alleles observed in some progenies, but not identified in the parents. This could be evidence of selection pressure, genomic rearrangements and/or alien introgression, as has been detected in strawberry (*Fragaria x ananassa*) [28] and wheat (*Triticum aestivum* L.) [29]. Another explanation might be that creating intergeneric hybrids induces severe genomic “shock” [30] characterised by widespread epigenetic changes across the genome. Such regions may result in highly compacted chromatin for one of the haplotypes, which means that the markers could not successfully hybridise for one of the alleles in such regions. Further research is required to identify the molecular causes of our observation that a significant portion of randomly distributed SNP markers do not support hybridity as expected. Nevertheless, from the results described here, it can be concluded that the SNP array technology is an appropriate approach for the genetic analysis of putative intergeneric hybrids, as many samples can be analysed in parallel, but this technology should not be used in isolation. We suggest that an array should constructed with a better balance of apple and pear sequence for future studies.

The predominance of maternal alleles in the hybrid genome has been observed previously in wide crosses. F1 progeny of *Brassica napus* L. × *Lesquerella fendleri* (A. Gray) S. Wats. mostly resembled female *B. napus* parents, based on AFLP (Amplified fragment length polymorphism) analysis [31]. However, some hybrids and their progenies were mixoploids with only 1–2 chromosomes, or chromosomal fragments of *L. fendleri* introgressed. Alleles absent in *B. napus* and specific for *L. fendleri* appeared in all F1 plants and their progenies. Progeny of interspecific crosses between annual sunflower (*Helianthus annuus* L.) and the wild perennial species *H. mollis* Lam. and *Helianthus orgyalis* DC. did not manifest the expected ratios of a true F1 hybrid, and in all cross configurations the progeny were non-Mendelian partial hybrids, with the female parent predominating [32]. In interspecific hybrids of *Momordica charantia* L. × *Momordica balsamina* L., SSR analysis showed that amplified fragments of interspecific hybrids exhibited highly conserved similarity with alleles of the female parents, with a very low frequency of male fragments Rathod *et al*. [33].

All these findings support our study, where screening with the 34 published apple and pear SSR markers (Supplementary Table 5) distributed over 15 of the 17 linkage groups (LGs), with one or more markers on each chromosome for apple or pear or both, suggested strongly that all the New Zealand CO progeny are closer to the maternal rather than to the paternal parent. The HRM analysis confirmed that the CO accessions were all hybrids, with 9–15 primers per genotype providing evidence for hybridity, with the apple female parent “Cox’s Orange Pippin” contributing more of its genome than the pear male parent. These results were further confirmed by the results from the SNP array.

When Polgári *et al*. [34] studied the composition and random elimination of paternal chromosomes in a large population of wheat × barley (*T. aestivum* L. × *Hordeum vulgare* L.) hybrids using a combination of DNA markers and genomic *in situ* hybridisation, analysis revealed an equal proportion of haploid and full hybrids between wheat and barley (20.5% and 19.5%, respectively), while the rest of the population contained hypoploids (partial hybrids) with no preference for any possible numbers (one to six) of barley paternal chromosome additions. However, we detected no whole chromosome elimination in our study, unlike these findings and the study of *B. napus* x *L. fendleri* progeny discussed above. This might be related to the comparatively simple ploidy status of the *Malus*-*Pyrus* cross; where both parents are diploid with the same chromosome number 2n = 34, while *B. napus* (2n = 38) is tetraploid and *L. fendleri* is diploid (2n = 12). Similarly, wheat (2n = 42) is hexaploid and barley (2n = 14) is diploid.

It is striking that the CO progeny inherited only the female parent’s, *Malus*-specific secondary metabolite phloridzin, indicating that the part of the apple maternal genome carrying genes controlling the biosynthesis of phloridzin was preferentially retained in the progeny. Similarly, the parents did not contribute equally to the hybrid genome in all the progeny of a second, pear x apple, cross, P265R232T018 x A199R45T055, where it was observed that seedling P26A19 3 synthesized only arbutin, while P26A19 4 synthesized both arbutin and phloridzin, indicating that the traits can be expressed simultaneously in progeny. Again, SSR markers demonstrated that the genomes of the P26A19 progeny are closer to those of the mother, pear P265R232T018, than the father, apple A199R45T055. Eight to 30 HRM markers per genotype confirmed that the parents did not contribute equally to the genomes of their progenies, with the maternal contribution predominating.

The predominance of the maternal contribution was also reported by Chen *et al*. [35] in progeny of *Brassica rapa*, and *B. napus* with *Capsella bursa-pastoris* (L.) Medik., where a majority of F1 plants resembled the female parents in morphology and only a few expressed some characters of the male parent. Parents’ uneven contribution to hybrid genomes is a likely outcome of incompatibility in intergeneric crosses [31, 36].

However, the pattern of the maternal genome predominating in the progeny was broken in the case of Zwintzscher’s Hybrid, which we had employed as a positive control for our marker studies. This hybrid is the product of a controlled pollination that was raised from a seed, as in the present study. The apple and pear parents contributed equally to this complete Mendelian hybrid, as shown by our SSR and HRM analyses and it accumulates both arbutin and phloridzin, as reported in Fischer *et al*. [12]. However the progeny of Zwintzscher’s Hybrid that resulted from open pollination accumulated only *Malus* specific [37] phloridzin. Our SSR and HRM analyses indicate strongly that the pollen parent(s) of these F2 were *Malus*, corroborated by the fact that in the field the Zwintzscher’s Hybrid trees were located adjacent to *Malus* accessions. In addition, phenotypic observations of the F2 progeny in the field showed they were more similar to apple than pear, with both the leaves and the fruit exhibiting a shape similar to *Malus*.

Ten SSR and four HRM markers provided evidence for hybridity of FP12 1, which accumulates phloridzin from the female parent rather than arbutin from the male parent. In contrast, the concentration of arbutin in the F2 OP progeny of this plant, F2-FP12 1–1.2-OP, was considerably higher than that of phloridzin, and leading to the conclusion that F2-FP12 1–1.2-OP was the result of pollination of FP12 1 by a pear, or that the second apple parent has contributed genes enhancing arbutin production. “Imperial Gala” and “Fuji” progenies produced only the maternal phloridzin genus-specific secondary metabolites (data not shown).

In the past, the presence of arbutin in pear has been correlated with the biochemical processes that operate as defence mechanisms against bacterial invasion. It has been suggested that the oxidation pathway of arbutin degradation may be involved in fire blight resistance of some pear varieties via the formation of substances toxic to the pathogen [4]. In the present study, the preliminary phenotyping of progeny for fire blight resistance indicated greater resistance in the intergeneric progeny than the parents (“Cox’s Orange Pippin” x “Old Home”). It is interesting to note that both the pear parents have fire blight resistance in their pedigrees and this might be expected to segregate in the progeny [21, 38]. These findings should be validated.

Work is also needed to map recombination events during the crossing of apple and pear more precisely along the chromosomes of the apple x pear hybrids, to identify which parental chromosomal region might be responsible for increased resistance. If confirmed, the resistant hybrids will provide an opportunity to develop apple-like hybrid cultivars with a pear fire blight resistance or a pear-like hybrid cultivar with apple fire blight resistance. A pear-like hybrid cultivar with apple fire blight resistance would also be a useful resource for pipfruit breeders developing fire blight resistant pears.

Detailed phenotyping of disease resistance in F2-FP12–1-1.2-OP and its parents, as well as the F2 progeny, would give useful data regarding their resistance or susceptibility to fire blight and maybe other diseases, such as apple and pear scab. This could be combined with precise and high resolution mapping of recombination events during the crossing of apple and pear, to accurately characterise the chromosomes of F2-FP12–1-1.2-OP, its parents, its F2 progeny and if possible the unknown male parent of the F2. Areas of strong segregation distortion might be expected in such genetic maps constructed in progeny derived from wide crosses [39]. We suggest that whole genome sequencing (WGS) is currently cost effective and less prone to artefacts than SNP array analysis, where there may be difficulties during hybridization of progeny DNA to the probes. In addition, WGS would provide more rigorous data concerning the parts of the genomes of pear or apple that are introgressed in the F2 progeny of F2-FP12–1-1.2-OP. A ploidy analysis would be interesting, in light of the chromosomal rearrangements observed in wide crosses between other species. Pedigree analysis could be used to identify the male parent of the F2 progeny that resulted from open pollination.

In conclusion, this research has demonstrated that HRM, SSR and SNP markers, combined with metabolomics, are very powerful tools to detect and confirm hybridity of progeny derived from crosses between apple and pear. This is particularly definitive when adequate numbers of these different marker types are used in combination to screen a progeny set. The non-Mendelian hybrids identified with the methods described here will be particularly suitable for advancement in breeding programmes, to introduce defined traits of interest, such as resistance, flavour or texture into either apple or pear.

## Materials and methods

### Plant material

The plant material used in the present study was from three different sources. FEM provided four members of the pedigree of Zwintscher’s Hybrid [12] (*M.* x *domestica* “Kalco” “Cox’s Orange Pippin”, and *P. communis* “André Desportes” as well as “Williams Bon Chretien”, also known as “Bartlett” or “Bon Chrétien”), Zwintzscher’s Hybrid, and five accessions of putative F2 progeny from open pollination of this hybrid [12]. Material held at PFR sites at Hawke’s Bay (HB) and Palmerston North (PN) included 95 putative F1 hybrids from nine different controlled crosses including “Cox’s Orange Pippin” x *P. communis* “Old Home”, (Supplementary Table 3), their parents and 29 F2 progeny from open pollination of putative F1 hybrids (Supplementary Table 4). The generation of PFR hybrids is described in Supplementary Methods.

### DNA isolation

Prior to SSR analysis of FEM accessions, DNA was extracted from fresh leaves using the NucleoSpin Plant II® Macherey Nagel kit. DNA quality and quantity were assessed with a NanoDrop™ 8000 Spectrophotometer (Thermo Scientific™). For the PFR samples used for SSR analysis, and FEM and PFR samples for HRM analysis, DNA was extracted from milled freeze-dried leaves using a modified cetyl-trimethylammonium bromide (CTAB) method [40]. The DNA quality and quantity were evaluated with a Qubit® 2.0 Fluorometer (Invitrogen, Life Technologies Corporation).

Prior to SNP array analysis, DNA was extracted from freeze-dried young leaves using the DNeasy® Plant Mini Kit (Qiagen, http://www.qiagen.com/) and the DNA quality and quantity were evaluated as above.

### S‌SR genotyping

Thirty-four published apple and pear SSR markers distributed over 15 of the 17 LGs (Supplementary Table 5) were selected to screen the PFR hybrids and their parents. PCR amplification was performed using the protocol reported by Knäbel *et* al. [41], incorporating a modified version of the M13 universal primer system. Samples were then multiplexed in a 124-μL reaction volume including 100 μL of water, 6 μL of PCR FAM amplification product, 6 μL of PCR Vic/Hex product, 6 μL of PCR NED product and 6 μL of PCR PET product held at 95°C for 5 min. Amplification products were analysed on an ABI 3500 Genetic Analyzer (Applied Biosystems). The raw data were processed using GeneMarker® v 2.2.0 software (© SoftGenetics, LLC.) to determine allele size in base pairs (bp).

To assess the genetic relationship among the five F2 progeny from open-pollinated Zwintzscher’s Hybrid, this accession and its grandparents, 30 published apple SSR markers mapping to 16 of the 17 LGs (Supplementary Table 6) were selected. PCR was performed as in Fischer *et al*. [12]. Amplification products were analysed on an ABI Prism 3130xl Genetic Analyzer sequencer (Applied Biosystems). The raw data were processed using GeneMapper 4.0 software (Applied Biosystems) to determine allele size in bp and a data matrix with allele sizes for each locus by sample was developed (data not shown). The “discrimination power at each locus for parent and progeny” (PD), which provides an estimate of the probability that two randomly sampled accessions of the study would be differentiated by their allelic profiles [42], was calculated as follows: PD = 1-PI (probability of identity (PI)) using GenAlEx v. 6.51b2 software [43]. Finally, the same software was used to analyse the SSR data, in order to assess genetic relationships among the hybrids in a progeny set.

### HRM-based SNP analysis

Identification of SNP variants that were unique to the apple parents used in this study and the published double haploid “Williams Bon Chretien” pear genome [8], respectively was performed by aligning merged whole genome short reads-based sequencing data for 34 apple accessions available in-house at PFR to the double haploid “Williams Bon Chrétien” pear genome [8] with Bowtie2 v2.0.0 [44]. The SAM files were converted to BAM format and variants were detected using bcftools v1.2. The search was for “apple” (aligned to pear) vs “pear” double haploid “Williams Bon Chrétien” pear genome. These unique variants were the basis for designing 155 HRM primer pairs positioned near both ends and the middle of all 17 LGs. Primer design was performed with the pear genome as a reference, using Primer 3 (ver. 4.1.0, https://primer3.ut.ee/) to obtain amplicons ranging between 50 and 120 bp in size (Supplementary Table 7). The amplicons from the apple-pear hybrids (heterozygous) were predicted to have lower melting points than the apple homozygotes or pear homozygotes, enabling identification of hybridity from inspection of melting curves. An initial trial to determine the efficiency of each of the 155 primer pairs was carried out using DNA from “Cox’s Orange Pippin”, “Old Home”, an equimolar mixture of DNA from both accessions, five putative hybrids, “Kalco”, “Williams Bon Chrétien” (pear), Zwintzscher’s Hybrid and one of its F2s obtained from open-pollination, and two mixtures of DNA from “Cox’s Orange Pippin” (apple) and “Williams Bon Chrétien” (pear), with 39 pairs of informative primers being employed to screen all DNA samples in the CO population.

As previously described for SSRs, for each locus present in each population the number of different alleles (Na), number of effective alleles (Ne), Shannon’s information index (I), observed heterozygosity (Ho) and expected heterozygosity (He) were estimated using GenAlEx v. 6.51b2 software [43]. PD was calculated as above. The data were analysed using the GenAlEx v. 6.51b2 [43] software to assess genetic relationships among the hybrids of a progeny, and the application of the Paetkau *et al*. [45] population assignment method. This method is based on the following population assignment test [46]:

Λ = Lh/Lmax,

where Lh is the likelihood of drawing that individual’s genotype from the population in which it was sampled, given the observed set of allele frequencies, and Lmax is the maximum such likelihood observed for this genotype in any population (i.e. the likelihood for the population to which the individual would be assigned in the assignment test). The population assignment result for a pair of populations is visualized in an assignment plot where the value on the X-axis is Lh for one population (e.g. population A), and that on the Y-axis is Lh for the other population (e.g. population B). This plot provides a visual representation of the degree of genetic separation among the populations and is an ideal way to assess the likely power of assignment tests graphically.

### SNP array genotyping

The apple and pear Infinium® II 9 K SNP array [47, 48] was used for SNP genotyping following the Infinium® HD Assay Ultra protocol, and scanned with the Illumina HiScan (Illumina Inc., San Diego, USA).

Data were analysed using Illumina’s GenomeStudio v 1.0 software Genotyping Module, setting a GenCall threshold of 0.15. The software automatically determines the cluster positions of the AA/AB/BB genotypes for each SNP and displays them in normalized graphs. SNPs were filtered for GenTrain score > 0.60 and 87% missing calls; additionally SNPs exhibiting the presence of null allele in the progeny or the parents were removed. The SNP 9 K array data were analysed by chromosome using the GenAlEx Software [43] to assess genetic relationships among progeny and parents.

### Targeted metabolite analysis

Freeze-dried newly expanding leaf samples (100 mg) from putative hybrids between apple and pear were weighed into a 15-mL plastic tube and extracted with 4 mL of 80% methanol [49] in three biological replicates. Samples were rotated with a vertical multi-function rotator for 20 min and sonicated for 30 min. After 48 h in the dark at 4°C, samples were centrifuged at 1000 x g and 4°C for 10 min. The resulting supernatants were collected and filtered through a 0.22-μm polyvinylidene difluoride (PVDF) filter. The targeted analysis of polyphenol compounds was performed using the liquid chromatography tandem mass spectrometry (LC–MS/MS) method, coupled with multiple reaction monitoring (MRM) quantification by a slightly modified method according to Vrhovsek *et al*. [49] and optimised for *Rosaceae* tissues, including the expected species-specific metabolites. Statistical variance analysis was performed on the data for quantity of phloridzin and arbutin, using the Fisher randomization test [12].

### Fire blight resistance screening

Up to eight replicate trees each of the 31 PFR putative hybrids with apple as female parent, as well as the apple parent/reference accessions (“Cox’s Orange Pippin”, “Fuji”, “Imperial Gala”, “Red Delicious”, “Robusta 5”, “Splendour” and two apple seedlings, including advanced selection A199R45T055) were grafted onto apple “Malling 9”. Similarly, trees of hybrids with pear as the female parent, as well as the pear parent/reference accessions (“Old Home”, “Williams Bon Chrétien” and five pear seedlings including advanced selection P265R23T018) were grafted onto *Pyrus calleryana* to produce plants for assessment of fire blight resistance.

Prior to inoculation, these trees were held in the greenhouse under a temperature regime of 25°C (day) and 20°C (night), with 80% relative humidity (RH) for 14 days. The plants were then acclimated to 26°C and 95% RH for inoculation by the cut-leaf method [50]. Actively growing shoots about 25 cm long were inoculated by cutting off 2/3 of the two youngest expanding leaves with scissors dipped in an aqueous buffer suspension of *E. amylovora* strain Ea236 at 1 x 10^9^ cfu/mL. The plants were maintained at 26°C and 95% RH for 7 days, then a further 30 days at 25°C and 80% RH. Disease progress was observed four times during the period from 12 to 37 days after inoculation. Disease extent was quantified by expressing necrosis length as a percentage of the total shoot length, both measured downwards from the point of inoculation. The mean percentage necrosis was then calculated for each genotype at each observation date and the AUDPC was calculated using the trapezoidal rule [51]. A non-linear scale was used for the percentage of necrosis to determine the degree of resistance/susceptibility to fire blight [52].

## Acknowledgements

This work was funded by The Autonomous Province of Trento, Italy (ADP) and a PhD fellowship to GM co-funded by FEM and PFR. We are grateful to Dr Lester Brewer and Richard Volz for performing intergeneric crosses in the field. We thank Dr Diego Micheletti (Fondazione Edmund Mach, Italy) for his assistance with the analysis of the SNP dataset from the array.

## Author Contributions

MAM, SEG, SM, VGMB and DC conceived and designed the study. CW raised the PFR hybrid progenies. GP conducted the experiments and analysed the data, with input from CW, LP, VGMB, SM and DC. GP and SEG wrote the manuscript with input from VGMB, LP, DC and SM. All authors read and approved the final manuscript.

## Data availability

All data were included in the paper and its Supplementary Materials published online.

## Conflicts of interest statement

The authors have no competing interests to declare.

## Supplementary data

Supplementary data is available at *Horticulture Research* online.

## Supplementary Material

Web_Material_uhac239
