# Supplementary material for: Molecular characterization of intergeneric hybrids between Malus and Pyrus
Source: Hortic Res. 2022 Oct 26;10(1):uhac239. doi: 10.1093/hr/uhac239 (PMC9832871; doi:10.1093/hr/uhac239)
Supplement: Web_Material_uhac239 [file web_material_uhac239.zip › Suppl_SEG_Pasqualetto 11-07-2022.docx]

**Supplementary Information**

**Supplementary Methods**

Hybrid progenies generated at PFR resulted from several rounds of controlled crossing in the field at HB (Richard Volz), Motueka (Lester Brewer) and at PN in the laboratory using controlled conditions to synchronize flowering. All crosses were performed with unmixed pollen and the direction of the cross varied. To simulate a dormancy phase, seeds were stored in darkness at 4°C for a minimum of four weeks and were then extracted. Seeds were extracted under sterile conditions and those fully developed were rinsed with a mixture of distilled water and commercial bleach (7:3 or 5:5 v:v), before being placed on tissue culture media #4 (full MS media with B5 vitamins and with low amounts of plant growth regulators (PGRs including GA3) reported by Debenham^52^ *et al.*) (Supplementary Fig. 5A). Developing plantlets were raised in tissue culture tubs until the 4-8 leaf stage and then placed on rooting medium #2873 (medium #2 of Pathirana *et al*.^53^, with Indole-3-butric acid 0.5 mg/L to enable root development (Supplementary Fig. 5B) before being transplanted into potting medium in a fog tunnel in the glasshouse (Supplementary Fig. 5C). After 1–14 days, plants were acclimatized in a temperature-controlled mist bed held at 24°C (+/-1°C), before moving progressively to a standard glasshouse (Supplementary Fig. 5D), shade house, outside in the PN nursery area (Supplementary Fig. 5E) and then to the research orchard at HB.

**
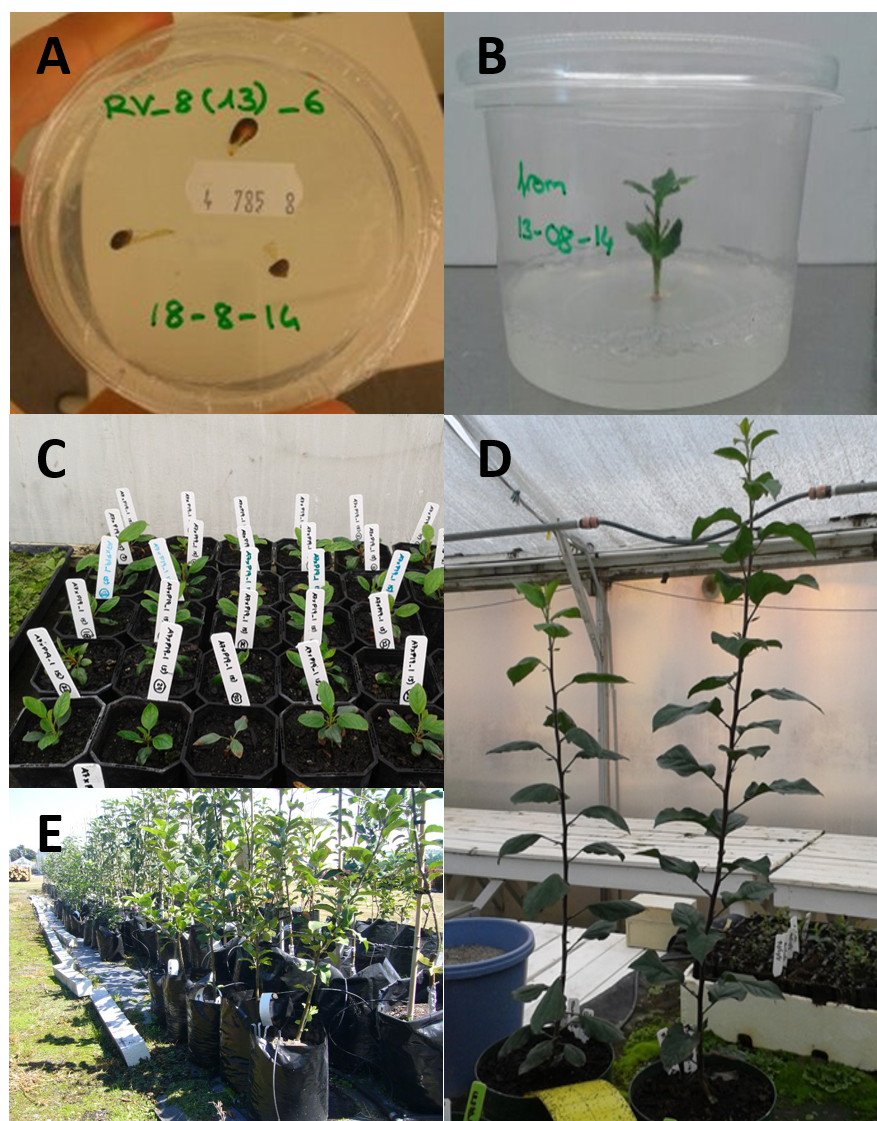
**

**Supplementary Fig. 5** A Germination of intergeneric apple/pear hybrid seeds on tissue culture medium. B. Developing plantlet in rooting medium. C. Rooted seedlings, potted up in fog tunnel. D. Potted up seedlings in glasshouse. E. Seedlings in nursery standing area at Palmerston North.

**Supplementary Results**

*Development of intergeneric crosses*

Seeds extracted from the mature fruits resulting from crosses made in both directions were not uniform in appearance. Some were plump and appeared fully developed, whilst others were thin and discarded as being probably unviable. Germination rate of apparently viable seeds was variable and was on average lower than seed from crosses within apple or pear, respectively. The development of roots was very poor across all plants and losses were extensive following deflasking from tissue culture media and transfer to potting mix.

**Supplementary References**

52 Debenham M, Seelye J, Horticultural AM. An in vitro repository for clonal kiwifruit. *XXIX Int Hortic Congr Hortic Sustain Lives, Livelihoods Landscapes* 2014; **1113**: 93–98.

53 Pathirana R, Mathew L, McLachlan A. A simplified method for high recovery of kiwifruit (Actinidia spp.) shoot tips after droplet vitrification cryopreservation suitable for long-term conservation. *Plant Cell Tissue Organ Cult* 2021; **144**: 97–102.

54 Yamamoto T, Kimura T, Shoda M, Ban Y, Hayashi T, Matsuta N. Development of microsatellite markers in the Japanese pear (Pyrus pyrifolia Nakai). *Mol Ecol Notes* 2002; **2**: 14–16.

55 Liebhard R, Gianfranceschi L, Koller B *et al.* Development and characterisation of 140 new microsatellites in apple (Malus x domestica Borkh.). *Mol Breed* 2002; **10**: 217–241.

56 Celton JM, Tustin DS, Chagné D, Gardiner SE. Construction of a dense genetic linkage map for apple rootstocks using SSRs developed from Malus ESTs and Pyrus genomic sequences. *Tree Genet Genomes* 2009; **5**: 93–107.

57 Vinatzer BA, Patocchi A, Tartarini S, Gianfranceschi L, Sansavini S, Gessler C. Isolation of two microsatellite markers from BAC clones of the Vf scab resistance region and molecular characterization of scab-resistant accessions in Malus germplasm*. *Plant Breed* 2004; **123**: 321–326.

58 Silfverberg-Dilworth E, Matasci CL, Van De Weg WE *et al.* Microsatellite markers spanning the apple (Malus x domestica Borkh.) genome. *Tree Genet Genomes* 2006; **2**: 202–224.

59 Guilford P, Prakash S, Zhu JM *et al.* Microsatellites in Malus X domestica (apple): Abundance, polymorphism and cultivar identification. *Theor Appl Genet* 1997; **94**: 249–254.

**Supplementary tables**

**Supplementary Table 1** Genetic diversity of simple sequence repeat (SSR) results for means of all the intergeneric apple/pear populations. Na (number of different alleles), Ne (number of effective alleles, I (Shannon's information index), Ho (observed heterozygosity), He (expected heterozygosity) and PD (discrimination power at each locus for parent and progeny). CO = ‘Cox’s Orange Pippin’ x ‘Old Home’; FEM = Fondazione Edmund Mach.

|  | Na | Ne | I | Ho | He | PD_Parent | PD_F1 |  | |
| --- | --- | --- | --- | --- | --- | --- | --- | --- | --- |
| CO | 1.5–4 | 1.3–3.06 | 0.28–1.13 | 0–0.99 | 0.19–0.64 | 0–0.89 | 0–0.82 |  | |
| P26A19 | 1.5–3 | 1.4–2.83 | 0.32–1.07 | 0–0.58 | 0.22–0.65 | 0–0.51 | 0–0.54 |  | |
|  | **Na** | **Ne** | **I** | **Ho** | **He** | **PD_Parent** | **PD_F1** | **PD_F2** |  |
| FEM | 1.67–4.33 | 1.38–3.82 | 0.3–1.28 | 0.08–1 | 0.18–0.68 | 0.72–0.96 | 0–0.63 | 0–0.95 |  |

**Supplementary Table 2** Genetic diversity of high resolution melting (HRM) results for means of all the intergeneric apple/pear populations. Na (number of different alleles), Ne (number of effective alleles, I (Shannon's information index), Ho (observed heterozygosity), He (expected heterozygosity) and PD (discrimination power at each locus for parent and progeny). CO = ‘Cox’s Orange Pippin’ x ‘Old Home’; FEM = Fondazione Edmund Mach.

|  | Na | Ne | I | Ho | He | PD_Parent | PD_F1 |  |
| --- | --- | --- | --- | --- | --- | --- | --- | --- |
| CO | 1.5–2.5 | 1.3–2.15 | 0.28–0.8 | 0–1 | 0.19–0.53 | 0.54–0.63 | 0–0.71 |  |
| P26A19 | 1.5–2.5 | 1.44–2.45 | 0.35–0.89 | 0–0.88 | 0.25–0.58 | 0.54–0.63 | 0–0.81 |  |
|  | **Na** | **Ne** | **I** | **Ho** | **He** | **PD_Parent** | **PD_F1** | **PD_F2** |
| FEM | 1.67–2 | 1.53–2 | 0.42–0.69 | 0–0.68 | 0.29–0.5 | 0.54–0.63 | 0–0.63 | 0–0.63 |

**Supplementary Table 3** Putative F1 hybrids from crosses between apple and pear or vice versa in New Zealand. The growth site is indicated as HB or PN for Hawke’s Bay and Palmerston North, respectively.

| Hybrid | Female |  | Male | Location |  | Hybrid | Female |  | Male | Location |
| --- | --- | --- | --- | --- | --- | --- | --- | --- | --- | --- |
| CO 1 | ‘Cox’s Orange Pippin’ | x | ‘Old Home’ | HB |  | **CO 27** | ‘Cox’s Orange Pippin’ | x | ‘Old Home’ | HB |
| CO 2 | ‘Cox’s Orange Pippin’ | x | ‘Old Home’ | HB |  | **CO 28** | ‘Cox’s Orange Pippin’ | x | ‘Old Home’ | HB |
| CO 3 | ‘Cox’s Orange Pippin’ | x | ‘Old Home’ | HB |  | **CO 29** | ‘Cox’s Orange Pippin’ | x | ‘Old Home’ | HB |
| CO 4 | ‘Cox’s Orange Pippin’ | x | ‘Old Home’ | HB |  | **CO 30** | ‘Cox’s Orange Pippin’ | x | ‘Old Home’ | HB |
| CO 5 | ‘Cox’s Orange Pippin’ | x | ‘Old Home’ | HB |  | **CO 31** | ‘Cox’s Orange Pippin’ | x | ‘Old Home’ | HB |
| CO 6 | ‘Cox’s Orange Pippin’ | x | ‘Old Home’ | HB |  | **CO 32** | ‘Cox’s Orange Pippin’ | x | ‘Old Home’ | HB |
| CO 7 | ‘Cox’s Orange Pippin’ | x | ‘Old Home’ | HB |  | **CO 33** | ‘Cox’s Orange Pippin’ | x | ‘Old Home’ | HB |
| CO 8 | ‘Cox’s Orange Pippin’ | x | ‘Old Home’ | HB |  | **CO 34** | ‘Cox’s Orange Pippin’ | x | ‘Old Home’ | HB |
| CO 9 | ‘Cox’s Orange Pippin’ | x | ‘Old Home’ | HB |  | **CO 35** | ‘Cox’s Orange Pippin’ | x | ‘Old Home’ | HB |
| CO 10 | ‘Cox’s Orange Pippin’ | x | ‘Old Home’ | HB |  | **CO 36** | ‘Cox’s Orange Pippin’ | x | ‘Old Home’ | HB |
| CO 11 | ‘Cox’s Orange Pippin’ | x | ‘Old Home’ | HB |  | **CO 37** | ‘Cox’s Orange Pippin’ | x | ‘Old Home’ | PN |
| CO 12 | ‘Cox’s Orange Pippin’ | x | ‘Old Home’ | HB |  | **CO 38** | ‘Cox’s Orange Pippin’ | x | ‘Old Home’ | PN |
| CO 13 | ‘Cox’s Orange Pippin’ | x | ‘Old Home’ | HB |  | **CO 39** | ‘Cox’s Orange Pippin’ | x | ‘Old Home’ | PN |
| CO 14 | ‘Cox’s Orange Pippin’ | x | ‘Old Home’ | HB |  | **CO 40** | ‘Cox’s Orange Pippin’ | x | ‘Old Home’ | PN |
| CO 15 | ‘Cox’s Orange Pippin’ | x | ‘Old Home’ | HB |  | **CO 41** | ‘Cox’s Orange Pippin’ | x | ‘Old Home’ | PN |
| CO 16 | ‘Cox’s Orange Pippin’ | x | ‘Old Home’ | HB |  | **P26A17** | P265R232T018 | x | Apple seedling | HB |
| CO 17 | ‘Cox’s Orange Pippin’ | x | ‘Old Home’ | HB |  | **P26A19 1** | P265R232T018 | x | A199R45T055 | HB |
| CO 18 | ‘Cox’s Orange Pippin’ | x | ‘Old Home’ | HB |  | **P26A19 2** | P265R232T018 | x | A199R45T055 | HB |
| CO 19 | ‘Cox’s Orange Pippin’ | x | ‘Old Home’ | HB |  | **P26A19 3** | P265R232T018 | x | A199R45T055 | HB |
| CO 20 | ‘Cox’s Orange Pippin’ | x | ‘Old Home’ | HB |  | **P26A19 4** | P265R232T018 | x | A199R45T055 | HB |
| CO 21 | ‘Cox’s Orange Pippin’ | x | ‘Old Home’ | HB |  | **FP12 1** | ‘Fuji’ | x | P125R095T02 | HB |
| CO 22 | ‘Cox’s Orange Pippin’ | x | ‘Old Home’ | HB |  | **FP12 2** | ‘Fuji’ | x | P125R095T02 | HB |
| CO 23 | ‘Cox’s Orange Pippin’ | x | ‘Old Home’ | HB |  | **FP12 3** | ‘Fuji’ | x | P125R095T02 | HB |
| CO 24 | ‘Cox’s Orange Pippin’ | x | ‘Old Home’ | HB |  | **FP12 4** | ‘Fuji’ | x | P125R095T02 | HB |
| CO 25 | ‘Cox’s Orange Pippin’ | x | ‘Old Home’ | HB |  | **FP12 5** | ‘Fuji’ | x | P125R095T02 | HB |
| CO 26 | ‘Cox’s Orange Pippin’ | x | ‘Old Home’ | HB |  |  |  |  |  |  |

**Supplementary Table 4** F2 hybrids from open pollination of putative F1 intergeneric apple/pear hybrids from New Zealand. The growth site is Hawke’s Bay (HB). Open pollination (OP).

| Hybrid | Female |  | Male | Location |  |
| --- | --- | --- | --- | --- | --- |
| F2-FP12 1-1.2-OP | FP12 1 | x | OP | HB |  |
| F2-FP12 1-1.3A-OP | FP12 1 | x | OP | HB |  |
| F2-FP12 1-1.5A-OP | FP12 1 | x | OP | HB |  |
| F2-FP12 1-1.6-OP | FP12 1 | x | OP | HB |  |
| F2-FP12 1-1.7-OP | FP12 1 | x | OP | HB |  |
| F2-FP12 1-1.8-OP | FP12 1 | x | OP | HB |  |
| F2-FP12 1-3.3-OP | FP12 1 | x | OP | HB |  |
| F2-FP12 1-3.4-OP | FP12 1 | x | OP | HB |  |
| F2-FP12 1-3.7-OP | FP12 1 | x | OP | HB |  |
| F2-FP12 1-3.8-OP | FP12 1 | x | OP | HB |  |
| F2-FP12 1-3.5-OP | FP12 1 | x | OP | HB |  |
| F2-FP12 1-1.11-OP | FP12 1 | x | OP | HB |  |
| F2-FP12 1-3.1-OP | FP12 1 | x | OP | HB |  |

**Supplementary Table 5** Details of the primers used for apple simple sequence repeat (SSR) marker analysis of the New Zealand Institute for Plant and Food Research Limited intergeneric apple/pear populations. LG indicates the linkage group and Tm the annealing temperature.

| Marker | LG | Tm (°C) | Forward primer sequence | Reverse primer sequence | Genus |
| --- | --- | --- | --- | --- | --- |
| NH013a^54^ | 1 | 57 | GGTTTGAAGAGGAATGAGGAG | CATTGACTTTAGGGCACATTTC | *Pyrus* |
| CH02b10^55^ | 2 | 57 | CAAGGAAATCATCAAAGATTCAAG | CAAGTGGCTTCGGATAGTTG | *Malus* |
| CH02c02a^55^ | 2 | 59 | CTTCAAGTTCAGCATCAAGACAA | TAGGGCACACTTGCTGGTC | *Malus* |
| NH002b^54^ | 2 | 58 | GGAGTCAGCGGCAAAAAAAG | CCCACTCCCTCCTCTTATTGT | *Pyrus* |
| CH02b12^55^ | 5 | 60 | GGCAGGCTTTACGATTATGC | CCCACTAAAAGTTCACAGGC | *Malus* |
| CH03a09^55^ | 5 | 62 | GCCAGGTGTGACTCCTTCTC | CTGCAGCTGCTGAAACTGG | *Malus* |
| TsuENH086^56^ | 5 | 61 | CTCTGTTCTGCTTCGATTCTGCT | GTTTCTTGTCCACGTTCACCATTTTTCAGT | *Pyrus* |
| CH04e05^55^ | 7 | 58 | AGGCTAACAGAAATGTGGTTTG | ATGGCTCCTATTGCCATCAT | *Malus* |
| CH01h10^55^ | 8 | 60 | TGCAAAGATAGGTAGATATATGCCA | AGGAGGGATTGTTTGTGCAC | *Malus* |
| NH029a^54^ | 9 | 62 | GAAGAAAACCAGAGCAGGGCA | CCTCCCGTCTCCCACCATATTAG | *Pyrus* |
| TsuENH008^56^ | 9 | 63 | CTGAGGTCTCATTCGGTGATTCT | GTTCTTCCTTCTCTGCTTTCTTCTTCACG | *Pyrus* |
| NH045a^56^ | 10 | 61 | ATCGAGAGACGAGGGTAGCA | TCTCTTGGCGTCTTCCTCTC | *Pyrus* |
| CH03d02^55^ | 11 | 60 | AAACTTTCACTTTCACCCACG | ACTACATTTTTAGATTTGTGCGTC | *Malus* |
| NB105a^56^ | 11 | 56 | AAACAACCGACTGAGCAACATC | AAAATCTTAGCCCAAAATCTCC | *Pyrus* |
| CH03c02^55^ | 12 | 59 | TCACTATTTACGGGATCAAGCA | GTGCAGAGTCTTTGACAAGGC | *Malus* |
| CH05d04^55^ | 12 | 59 | ACTTGTGAGCCGTGAGAGGT | TCCGAAGGTATGCTTCGATT | *Malus* |
| KA16^54^ | 12 | 56 | GCCAGCGAACTCAAATCT | AACGAGAACGACGAGCG | *Pyrus* |
| CH02g01^55^ | 13 | 60 | GATGACGTCGGCAGGTAAAG | CAACCAACAGCTCTGCAATC | *Malus* |
| NH021a^54^ | 13 | 58 | ATCTCAATTTTCTCGGTAACCA | CTGATATCTCTCTGCACTCCCT | *Pyrus* |
| CH01g05^55^ | 14 | 60 | CATCAGTCTCTTGCACTGGAAA | GACAGAGTAAGCTAGGGCTAGGG | *Malus* |
| CH03g06^55^ | 14 | 54 | ATCCCACAGCTTCTGTTTTTG | TCACAGAGAATCACAAGGTGGA | *Malus* |
| NH004a^54^ | 14 | 59 | AGGATGGGACGAGTTTAGAG | CCACATCTCTCAACCTACCA | *Pyrus* |
| TsuENH058^56^ | 14 | 61 | AGAAGAAGGATAAGAAGAAGGATGG | GTTTCTTGTAACGAAAAGGAAACAGGACTTG | *Malus* |
| CH02c09^55^ | 15 | 60 | TTATGTACCAACTTTGCTAACCTC | AGAAGCAGCAGAGGAGGATG | *Malus* |
| CH02d11^55^ | 15 | 60 | AGCGTCCAGAGCAACAGC | AACAAAAGCAGATCCGTTGC | *Malus* |
| NH027a^54^ | 15 | 56 | TAATGTGTTGGGGAGAGAGAG | GCTCTTGTTCCTTGCTCCTAA | *Pyrus* |
| CH05a04^55^ | 16 | 57 | GAAGCGAATTTTGCACGAAT | GCTTTTGTTTCATTGAATCCCC | *Malus* |
| CH01b12^55^ | 17 | 59 | CGCATGCTGACATGTTGAAT | CGGTGAGCCCTCTTATGTGA | *Malus* |
| CH04c10^55^ | 17 | 56 | GGGTTAGGTTGTCTTCTCTCCT | GCTTCTCGGGTGAGTTTTTC | *Malus* |
| Ch-Vf1^57^ | 1, 10 | 58 | ATCACCACCAGCAGCAAAG | CATACAAATCAAAGCACAACCC | *Malus* |
| CH04c06^55^ | 10, 17 | 60 | GCTGCTGCTGCTTCTAGGTT | GCTTGGAAAAGGTCACTTGC | *Malus* |
| TsuENH004^56^ | 4, 12 | 59 | CGCATTAAAGTCTGGCTTTCTTC | GAATTGGCAGAGAGATTGAGTGG | *Malus* |
| CH05a02^55^ | 8, 15 | 60 | GTTGCAAGAGTTGCATGTTAGC | TTTTGACCCCATAAAACCCAC | *Malus* |
| CH01h02^55^ | 9, 17 | 62 | AGAGCTTCGAGCTTCGTTTG | ATCTTTTGGTGCTCCCACAC | *Malus* |

**SupplementaryTable 6** Details of the primers used for apple simple sequence repeat (SSR) marker analysis of the Fondazione Edmund Mach intergeneric apple/pear populations. LG indicates the linkage group and Tm the annealing temperature.

| Marker | LG | Tm (°C) | Forward primer sequence | Reverse primer sequence |
| --- | --- | --- | --- | --- |
| Hi21g05^58^ | 1 | 59 | GACGAGCTCAAGAAGCGAAC | GTTTGCTCTTGCCATTTTCTTTCG |
| CH02f06^55^ | 2 | 59 | CCCTCTTCAGACCTGCATATG | ACTGTTTCCAAGCGATCAGG |
| CH02c02a^55^ | 2 | 57 | CAAGGAAATCATCAAAGATTCAAG | CAAGTGGCTTCGGATAGTTG |
| Ch03g07^55^ | 3 | 57 | AATAAGCATTCAAAGCAATCCG | TTTTTCCAAATCGAGTTTCGTT |
| MS14h03^55^ | 3 | 55 | CGCTCACCTCGTAGACGT | ATGCAATGGCTAAGCATA |
| CH02h11a^55^ | 4 | 57 | CGTGGCATGCTTATCATTTG | CTGTTTGAACCGCTTCCTTC |
| NZ05g08^59^ | 4 | 59 | CGGCCATCGATTATCTTACTCTT | GGATCAATGCACTGAAATAAACG |
| CH02a08z^55^ | 5 | 55 | GAGGAGCTGAAGCAGCAGAG | ATGCCAACAAAAGCATAGCC |
| Hi09B04^58^ | 5 | 59 | GCGATGACCAATCTCTGAAAC | TGGGCTTGAATTGGTGAATC |
| CH05a05^55^ | 6 | 55 | TGTATCAGTGGTTTGCATGAAC | GCAACTCCCAACTCTTCTTTCT |
| CH01c06^55^ | 8 | 59 | AAACTTTCACTTTCACCCACG | ACTACATTTTTAGATTTGTGCGTC |
| Hi04b12^58^ | 8 | 59 | CCCAAACTCCCAACAAAGC | GTTTGAGCAGAGGTTGCTGTTGC |
| CH01h02^55^ | 9 | 57 | AAACTTTCACTTTCACCCACG | ACTACATTTTTAGATTTGTGCGTC |
| CH05c07^55^ | 9 | 57 | TGATGCATTAGGGCTTGTACTT | GGGATGCATTGCTAAATAGGAT |
| Hi22f04^58^ | 10 | 59 | TCAATCCTCTGCTCTTCAAGG | GTTTAATCACCTGCTGCTGCTTG |
| MS06g03^55^ | 10 | 59 | CGGAGGGTGTGCTGCCGAAG | GCCCAGCCCATATCTGCT |
| CH04h02^55^ | 11 | 59 | GGAAGCTGCATGATGAGACC | CTCAAGGATTTCATGCCCAC |
| CH05d11^55^ | 11 | 57 | CACAACCTGATATCCGGGAC | GAGAAGGTCGTACATTCCTCAA |
| CH03c02^55^ | 12 | 55 | TCACTATTTACGGGATCAAGCA | GTGCAGAGTCTTTGACAAGGC |
| CH03a08^55^ | 13 | 57 | AAACTTTCACTTTCACCCACG | ACTACATTTTTAGATTTGTGCGTC |
| CH03b10^55^ | 13 | 57 | CCCTCCAAAATATCTCCTCCTC | CGTTGTCCTGCTCATCATACTC |
| AJ000761^58^ | 14 | 55 | CTGGGTGGATGCTTTGACTT | TCAATGACATTAATTCAACTTACAAAA |
| CH03d08^55^ | 14 | 55 | CATCAGTCTCTTGCACTGGAAA | TAGGGCTAGGGAGAGATGATGA |
| CH04c07^55^ | 14 | 59 | GGCCTTCCATGTCTCAGAAG | CCTCATGCCCTCCACTAACA |
| CH02d11^55^ | 15 | 55 | AGCGTCCAGAGCAACAGC | AACAAAAGCAGATCCGTTGC |
| NZ02b1^59^ | 15 | 59 | AAGAGGGTGTTCCCAGATCC | TGTTCGATGTGACTTCAATGC |
| CH05a04^55^ | 16 | 59 | GAAGCGAATTTTGCACGAAT | GCTTTTGTTTCATTGAATCCCC |
| CH05c06^55^ | 16 | 57 | ATTGGAACTCTCCGTATTGTGC | ATCAACAGTAGTGGTAGCCGGT |
| CH01h01^55^ | 17 | 55 | GAAAGACTTGCAGTGGGAGC | GGAGTGGGTTTGAGAAGGTT |
| CH05g03^55^ | 17 | 59 | GCTTTGAATGGATACAGGAACC | CCTGTCTCATGGCATTGTTG |

**Supplementary Table 7** Details of primers used for high resolution melting (HRM) analysis of the intergeneric apple/pear hybrids.

| Name | Genome location | SNP | Forward primer sequence | Reverse primer sequence | Expected amplicon size (bp) | Melting temperature (ºC) |
| --- | --- | --- | --- | --- | --- | --- |
| 1 | Chr1: 4650907-4651009 | 4650907 | GTCAATGCAACCTATCGCCA | TTAGTCACTGTGGGAAACGC | 103 | 55 |
| 2 | Chr1: 38997511-38997627 | 38997511 | CTCTAGCATGCAAAGGAACCC | TGGCACCATACACATACAATGA | 96 | 55 |
| 3 | Chr2: 306135-306226 | 306135 | GTGTTACTCCCTACATTGCCA | GTTTGACTTCACACACGGGA | 92 | 55 |
| 4 | Chr2: 14077723-14077841 | 14077723 | TCAGGTCTTGACAGGAAGGT | TCTTTGTCCCTTACCCTAGAGT | 119 | 55 |
| 5 | Chr2: 22240468-22240585 | 22240468 | TTGTTTCTTCGGCCATGGTG | CCGTCGAATTTGAAGCCCTT | 118 | 55 |
| 6 | Chr3: 3468716-3468771 | 3468716 | TGGTGTCAGCAATAACATACCG | TTCACAGCACATCCTTCTGC | 56 | 55 |
| 7 | Chr3: 6077512-6077630 | 6077512 | TGGCCACGGCACCTTATG | GGATTCGGTTTCTTCTTCTCTCAC | 119 | 55 |
| 8 | Chr4: 10571099-10571175 | 10571099 | GGGATGCAAAGTGACAATGG | TTCGTCAACACTTTGCATCTG | 77 | 55 |
| 9 | Chr4: 27271699-27271789 | 27271699 | TCACCAATCCACTCCTGTCA | GGCAAGAAATGAGGCACACA | 91 | 55 |
| 10 | Chr5: 4426301-4426418 | 4426301 | AAATCTCTGCCAGCAAAGAGG | GCTTGGCCAACTATCCCAAC | 118 | 55 |
| 11 | Chr5: 8856650-8856736 | 8856650 | ACAGCTCGAATCGATGTTCTG | GAGATCGAGAGCGTTGAAGC | 87 | 55 |
| 12 | Chr5: 23625942-23625997 | 23625942 | CGGAAGACTTCACACAAGCC | CATGCCTATGGATCCAAAAGC | 56 | 55 |
| 13 | Chr6: 1400544-1400660 | 1400544 | CTGCTTCTCTGTACGTCCTTC | GGAAGACGTATATTCACTTGAAGC | 117 | 55 |
| 14 | Chr6: 17485718-17485793 | 17485718 | CACTCTAGCCCAACGAACCT | TCTCGAGGTGTGGATGTAGC | 76 | 55 |
| 15 | Chr6:23002193-23002247 | 23002193 | TTAGAAGGATCGCCAGCAAC | TGAGCTCAAGGTATCTCCACC | 55 | 55 |
| 16 | Chr7: 3410996-3411051 | 3410996 | ACATTGTGATCAGGGTCTTCC | ACCACCAAGAACCTCAGCAT | 56 | 55 |
| 17 | Chr7: 20289452-20289564 | 20289452 | CATTCTGCAGTCACACCAAGA | TGCGGTTGATACAGTTCATCA | 113 | 55 |
| 18 | Chr8: 344642-344754 | 344642 | ACTCTCATTCCACTCCACCA | TGTGTCCCAAATGCATACGT | 113 | 55 |
| 19 | Chr8: 16391169-16391286 | 16391169 | GTTGAGCCATGATCTGCCTG | TGGAAGGCCATGCTTGAATG | 118 | 55 |
| 20 | Chr9: 2196631-2196718 | 2196631 | CAGCAGAATCGTCAAGAGCC | GATGTTCCAGGTTGAGCTGT | 88 | 55 |
| 21 | Chr9: 5441428-5441547 | 5441428 | GGGCCTGTACCAAACAAATTG | TGGTTGATGCTTGAAGAATGTTC | 120 | 55 |
| 22 | Chr10: 2643959-2644012 | 2643959 | TCTCTTGCTTGGTTCATGTCG | TTGACCAATGGTGACGAAGC | 54 | 55 |
| 23 | Chr10: 10168201-10168317 | 10168201 | CATCAAGACCAAGCTTCTCGG | TCAGGCAGGCTACTAAAGAGG | 117 | 55 |
| 24 | Chr11: 463545-463660 | 463545 | GTCAGCCGGCTCTTTATTCG | TGGTTCAAGCAGAGCAGC | 116 | 55 |
| 25 | Chr11: 23428118-23428212 | 23428118 | GCCGACCTATCAACCCAGTT | AGCTCTCGTAATCAGTGGCT | 95 | 55 |
| 26 | Chr12: 925714-925782 | 925714 | CACGACATTGACAAGGTGGA | AGGGGAACTATGAACAGCCA | 69 | 55 |
| 27 | Chr12: 6731573-6731673 | 6731573 | TCCTGCATCGTAAACATAGGTTC | TGCCGGCTTTCTGTTGATTC | 101 | 55 |
| 28 | Chr12: 20068163-20068254 | 20068163 | CCTTTACAAACGCTGACCTGT | AGCAGCAGAGTTCTCAGGTT | 92 | 55 |
| 19 | Chr13: 8624037-8624123 | 8624037 | GATATATTTCACCACCTCATGCAG | TCCGTGGAAAGTAAATATTGTAGC | 87 | 55 |
| 30 | Chr13: 26326450-26326524 | 26326450 | CATCCGTTAAATGGTGGGACA | ACAGAGACAACCCTTCCACA | 75 | 55 |
| 31 | Chr14: 2857003-2857097 | 2857003 | TGGGCGTCAAACTTCAATGA | ACTATGCCATGGTTTTACTCGT | 95 | 55 |
| 32 | Chr14: 19912302-19912416 | 19912302 | AGGCTAAACTCATAAACGTGTGG | CCACAAACCTCAGGCCATTC | 115 | 55 |
| 33 | Chr14:21391636-21391742 | 21391636 | CATGAATATGCTCTTTCTCTGATGG | CAGTGAAACAGCTGAGGGTG | 107 | 55 |
| 34 | Chr15: 326327-326425 | 326327 | AGCTGCAAATTGTCACAAGC | GAAACACTGAAGAGGCGAGG | 99 | 55 |
| 35 | Chr15: 773670-773764 | 773670 | CCATACCTATGCTCACGCTC | ATGGCTCCATTTCTGTCCTG | 95 | 55 |
| 36 | Chr15:25892571-25892671 | 25892571 | ACAATGGTGCTGACTGTGTT | TCCACAACTGCGAGACAATC | 101 | 55 |
| 37 | Chr16: 823477-823583 | 823477 | TCGAACGAGGACTGGGATG | AGGCATGTCTATCAAGCGTG | 107 | 55 |
| 38 | Chr16: 17140174-17140230 | 17140174 | TGAACTGTTGAACGAGGAGTC | AAAGCTGCATCTGTCGGAAG | 57 | 55 |
| 39 | Chr16: 29098000-29098108 | 29098000 | ATCTGGAGTAGCCCACGAAC | GTAAGTTCATAGTGAGGGCTGAC | 109 | 55 |

**Supplementary Figure Legends**

**Supplementary Fig. 1** Population assignment of putative intergeneric apple/pear hybrids as deduced from the single nucleotide polymorphism (SNP) chip analysis. The plots represent the positive log-likelihood of assignment of each sample by GenAlEx. The log-likelihood values calculated assigned the apple parent (‘Cox’s Orange Pippin’) to one group/population, the pear parent (‘Old Home’) to a second group/population and the putative hybrids to another. Figure 1A shows the analysis for SNP markers from LG1, 1B shows LG2, 1C LG3, and 1D LG4.

**Supplementary Fig. 5** A Germination of intergeneric apple/pear hybrid seeds on tissue culture medium. B. Developing plantlet in rooting medium. C. Rooted seedlings, potted up in fog tunnel. D. Potted up seedlings in glasshouse. E. Seedlings in nursery standing area at Palmerston North.

**Supplementary Fig. 4** Population assignment of putative intergeneric apple/pear hybrids as deduced from the single nucleotide polymorphism (SNP)-chip array analysis. The plots represent the positive log-likelihood of assignment of each sample by GenAlEx. The log-likelihood values calculated assigned the apple parent (‘Cox’s Orange Pippin’) to one group/population, the pear parent (‘Old Home’) to a second group/population and the putative hybrids to another. Figure 2A shows the analysis for SNP markers from LG5, 2B shows LG6, 2C LG7, and 2D LG8.

**Supplementary Fig. 3** Population assignment of putative intergeneric apple/pear hybrids as deduced from the single nucleotide polymorphism (SNP) chip array analysis. The plots represent the positive log-likelihood of assignment of each sample by GenAlEx. The log-likelihood values calculated assigned the apple parent (‘Cox’s Orange Pippin’) to one group/population, the pear parent (‘Old Home’) to a second group/population and the putative hybrids to another. Figure 3A shows the analysis for SNP markers from LG9, 3B shows LG10, 3C LG11, and 3D LG12.

**Supplementary Fig. 2** Population assignment of putative intergeneric apple/pear hybrids as deduced from the single nucleotide polymorphism (SNP) chip array analysis. The plots represent the positive log-likelihood of assignment of each sample by GenAlEx. The log-likelihood values calculated assigned the apple parent (‘Cox’s Orange Pippin’) to one group/population, the pear parent (‘Old Home’) to a second group/population and the putative hybrids. Figure 4A shows the analysis for SNP markers from LG13, 4B shows LG14, 4C LG15, 4D LG16, and 4E LG17.
