# Supplementary figures and images for: Molecular characterization of intergeneric hybrids between Malus and Pyrus
Source: Hortic Res. 2022 Oct 26;10(1):uhac239. doi: 10.1093/hr/uhac239 (PMC9832871; doi:10.1093/hr/uhac239)

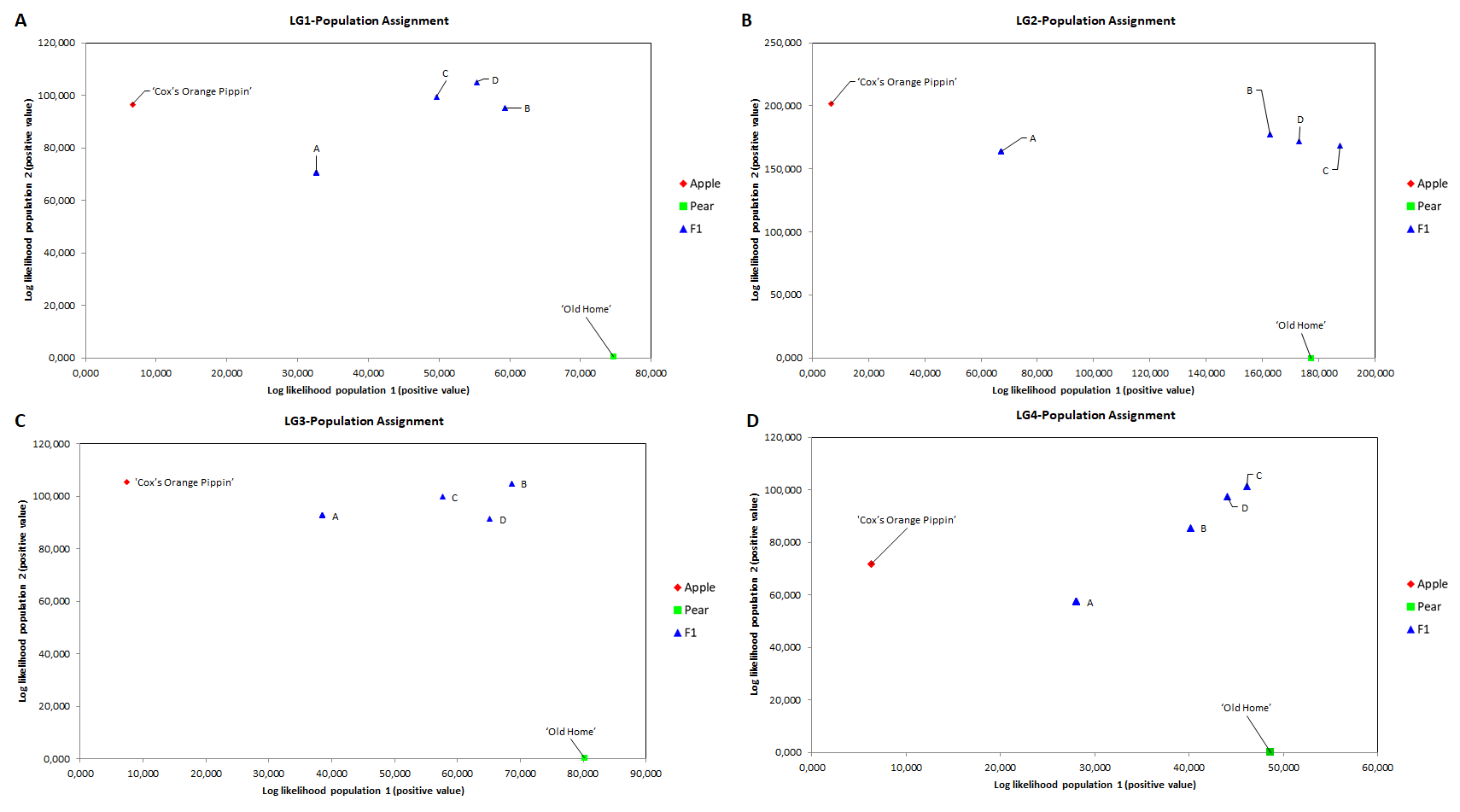

Supplement: Web_Material_uhac239 [file web_material_uhac239.zip › Suppl_Fig1.tif]

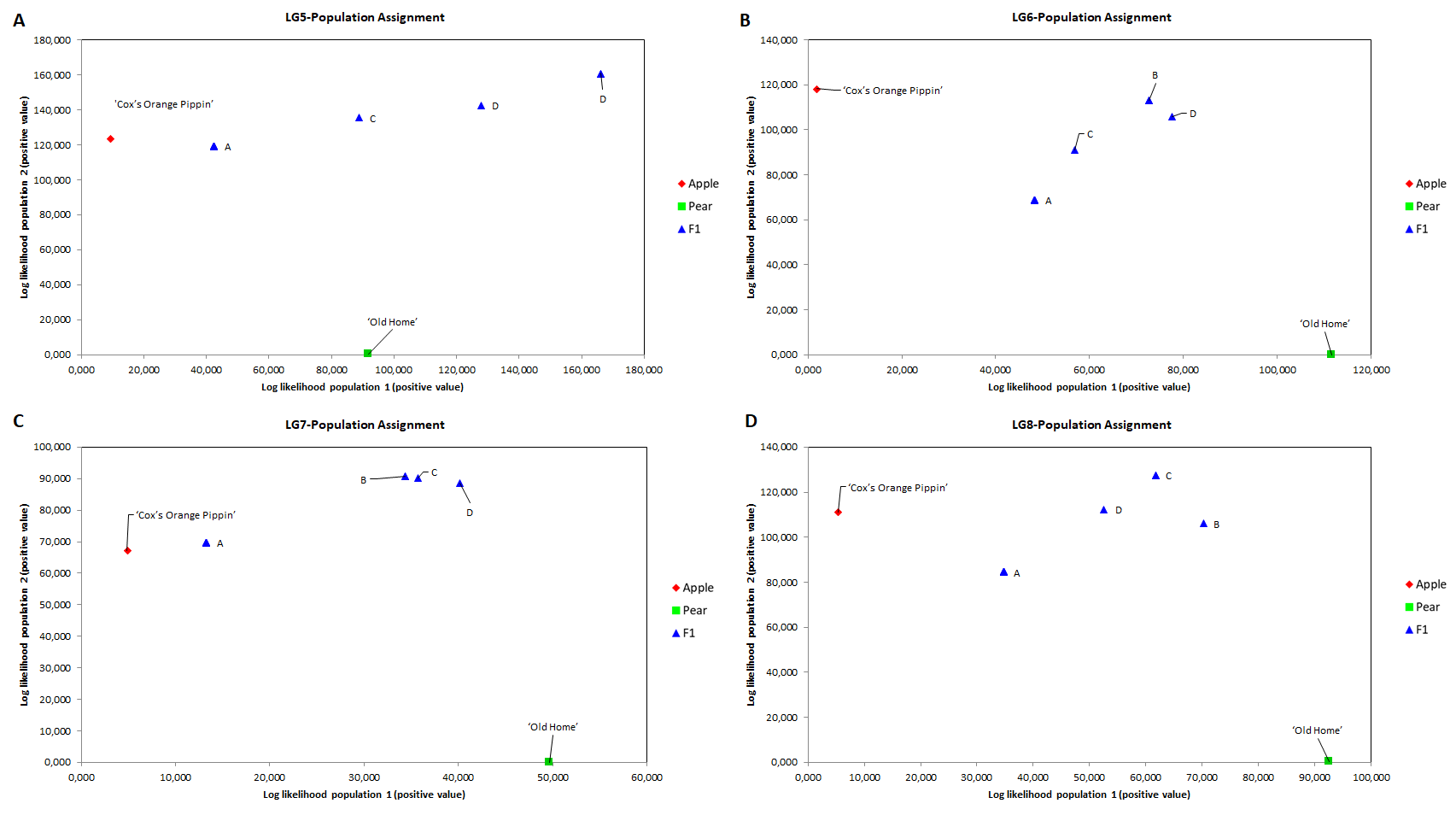

Supplement: Web_Material_uhac239 [file web_material_uhac239.zip › Suppl_Fig2.tif]

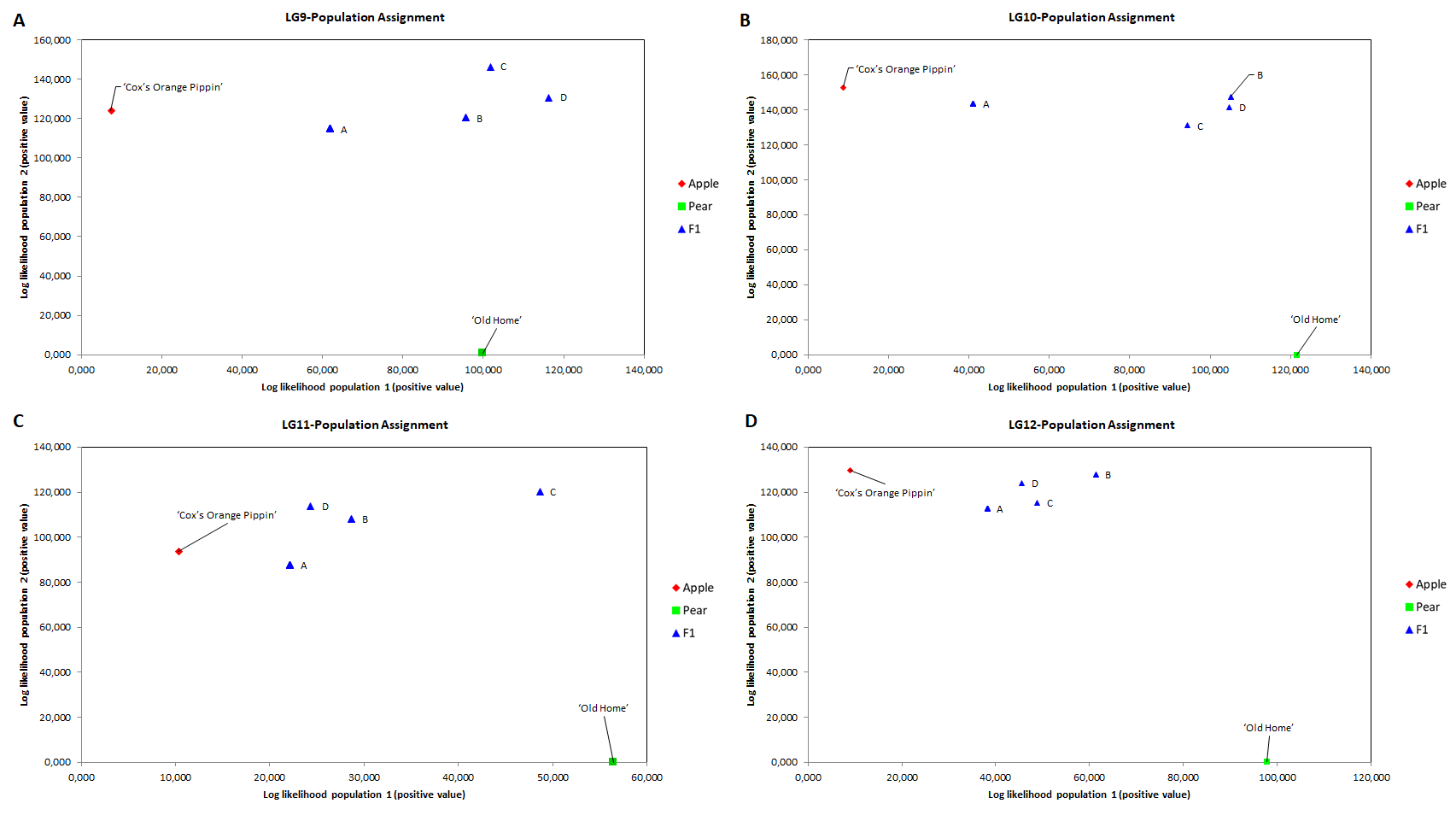

Supplement: Web_Material_uhac239 [file web_material_uhac239.zip › Suppl_Fig3.tif]

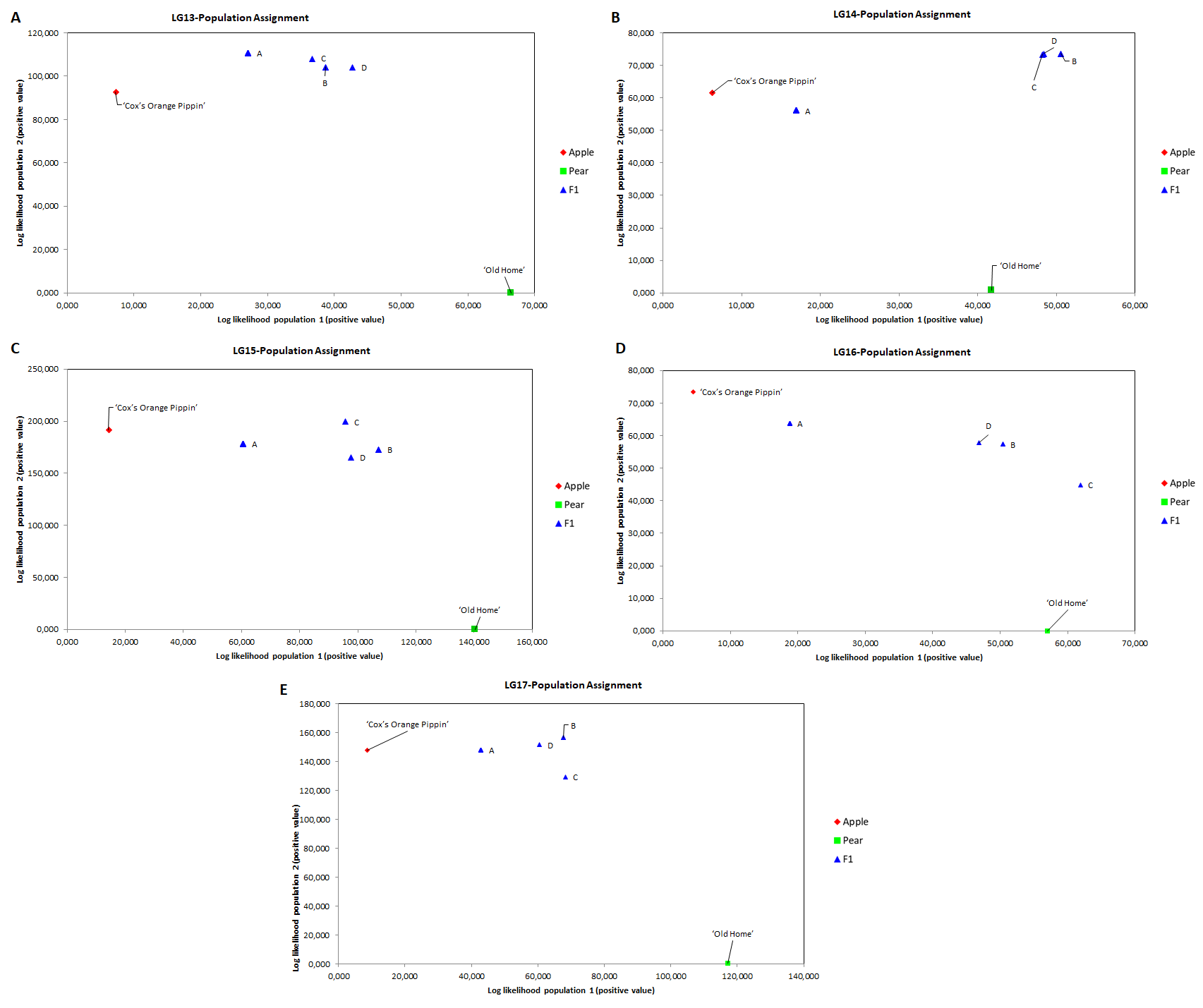

Supplement: Web_Material_uhac239 [file web_material_uhac239.zip › Suppl_Fig4.tif]
